# Intra-urban spatial variability of breast and cervical cancer mortality in the city of São Paulo: analysis of associated factors

**DOI:** 10.1590/1980-549720230008.2

**Published:** 2023-01-09

**Authors:** Breno Souza de Aguiar, Alessandra Cristina Guedes Pellini, Elizabeth Angélica Salinas Rebolledo, Adeylson Guimarães Ribeiro, Carmen Simone Grilo Diniz, Patricia Marques Moralejo Bermudi, Marcelo Antunes Failla, Oswaldo Santos Baquero, Francisco Chiaravalloti-Netto

**Affiliations:** I Secretaria Municipal da Saúde de São Paulo, Coordenação de Epidemiologia e Informação – São Paulo (SP), Brasil.; II Universidade Nove de Julho – São Paulo (SP), Brasil.; III Universidade de São Paulo, Faculdade de Saúde Pública – São Paulo (SP), Brasil.; IV Hospital de Câncer de Barretos, Instituto de Ensino e Pesquisa – Barretos (SP), Brasil.; V Universidade de São Paulo, Faculdade de Medicina Veterinária e Zootecnia – São Paulo (SP), Brasil.

**Keywords:** Breast neoplasm, Uterine cervical neoplasm, Mortality, Spatial regression, Câncer de mama, Câncer do colo do útero, Mortalidade, Regressão espacial

## Abstract

**Objective:**

To identify spatial variability of mortality from breast and cervical cancer and to assess factors associated in the city of São Paulo.

**Methods:**

Between 2009 and 2016, 10,124 deaths from breast cancer and 2,116 deaths from cervical cancer were recorded in the Mortality Information System among women aged 20 years and over. The records were geocoded by address of residence and grouped according to Primary Health Care coverage areas. A spatial regression modeling was put together using the Bayesian approach with a Besag-York-Mollié structure to verify the association of deaths with selected indicators.

**Results:**

Mortality rates from these types of cancer showed inverse spatial patterns. These variables were associated with breast cancer mortality: travel time between one and two hours to work (RR – relative risk: 0.97; 95%CI – credible interval: 0.93–1.00); women being the head of the household (RR 0.97; 95%CI 0.94–0.99) and deaths from breast cancer in private health institutions (RR 1.04; 95%CI 1.00–1.07). The following variables were associated with mortality from cervical cancer: travel time to work between half an hour and one hour (RR 0.92; 95%CI 0.87–0.98); per capita household income of up to 3 minimum wages (RR 1.27; 95%CI 1.18–1.37) and ratio of children under one year of age related to the female population aged 15 to 49 years (RR 1.09; 95%CI 1.01–1.18).

**Conclusion:**

The predicted RR for mortality from these cancers were calculated and associated with the socioeconomic conditions of the areas covered.

## RESUMO

**Objetivo:**

Identificar a variabilidade espacial da mortalidade por câncer de mama e colo do útero e avaliar fatores associados à mortalidade por esses cânceres no município de São Paulo.

**Métodos:**

Entre 2009 e 2016 foram registrados, no Sistema de Informações sobre Mortalidade, 10.124 óbitos por câncer de mama e 2.116 óbitos por câncer do colo do útero em mulheres com 20 anos e mais. Os registros foram geocodificados por endereço de residência e agregados segundo território adstrito. Foram realizadas modelagens de regressão espacial utilizando-se a abordagem bayesiana com estrutura de Besag-York-Mollié para verificar a associação dos óbitos com indicadores selecionados.

**Resultados:**

As taxas de mortalidade por esses cânceres apresentaram padrões espaciais inversos. As variáveis associadas à mortalidade por câncer de mama foram: tempo de deslocamento para o trabalho entre uma e duas horas (risco relativo — RR 0,97; intervalo de credibilidade — IC95% 0,93–1,00); mulheres responsáveis pelo domicílio (RR 0,97; IC95% 0,94–0,99) e óbitos por câncer de mama ocorridos em estabelecimentos privados (RR 1,04; IC95% 1,00–1,07). À mortalidade por câncer do colo do útero, estiveram associados: tempo de deslocamento para o trabalho entre meia e uma hora (RR 0,92; IC95% 0,87–0,98); rendimento domiciliar até três salários-mínimos (RR 1,27; IC95% 1,18–1,37); e razão de menores de um ano em relação à população feminina de 15 a 49 anos (RR 1,09; IC95% 1,01–1,18).

**Conclusão:**

Foram calculados os RR preditos para a mortalidade por esses cânceres, que estiveram associados às condições socioeconômicas das áreas de abrangência.

## INTRODUCTION

Breast cancer (BC) and cervical cancer (CC) are serious public health problems worldwide. Regarding mortality, in 2020, BC was the leading cause of death from cancer among women, totaling 17,825 deaths, while CC ranked fourth with 6,627 deaths^1^.

However, BC and CC profiles are marked by their inverse nature, with the presence of higher BC incidence rates, but not mortality rates, in higher-income countries, and higher CC incidence and mortality rates in countries with lower levels of Human Development Index^2^.

The high rate of BC in highly developed countries is attributed to the higher prevalence of risk factors, such as late motherhood, nulliparity, non-breastfeeding or short-term breastfeeding, early age menarche, menopause at older ages, use of oral contraceptives and hormone replacement, alcohol intake, overweight, diet rich in processed foods and red meats, physical inactivity, work shifts, unemployment having worked before, exposure to ionizing radiation such as mammography and radiotherapy and to endocrine disrupting chemicals^3–8^.

As for CC, the main risk factor is persistent infection with the human papillomavirus (HPV), which is responsible for almost all cases of this cancer, and types 16 and 18 cause approximately 70% of all cases^9^. Given that the route of transmission of HPV is sexual^10^, the risk factors increase with difficulties in accessing prevention measures and the characteristics of one’s sexual life (early start of sexual life, non-use of condoms, high number of sexual partners). Factors that help in the progression of infection to this cancer are high parity (>4 children), history of abortions, HIV immunosuppression, use of oral contraceptives, smoking, history of sexually transmitted infections (STIs), family history of CC, and menopause^11–16^. However, the knowledge on how geographic or temporal variations in morbidity and mortality from these types of cancer are related to specific etiological factors is still limited^2^.

For both BC and CC, there are well-designed preventive policies in place^17,18^; however, few studies have performed spatial data analysis^19–21^, especially at the intra-municipal level^22^. In the city of São Paulo, mortality from BC and CC show an inverse behavior, the former being more frequent in populations with better socioeconomic status, while the latter occurs in economically disadvantaged populations^23^. Because of the large municipal socioeconomic gradient, a spatial analysis of mortality from these diseases would make it possible to identify intra-urban spatial variability and associated factors, being an important tool to guide public policies.

## METHODS

**Figure 1.**
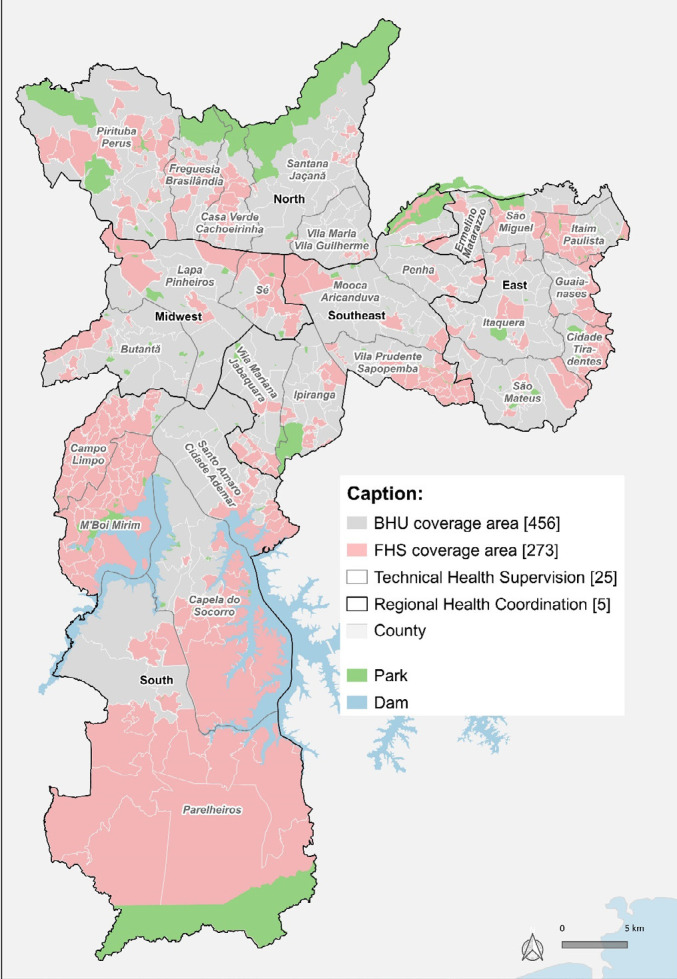
Areas covered by Basic Health Units (2015/2016) in the city of São Paulo.

BHU: Basic Health Unit; FHS: Family Health Strategy. Source: GISA/CEInfo/SMS-SP (version 2015/2016).

The city of São Paulo, capital of São Paulo State, is its largest, with estimated resident population in 2017 of 11,696,088 inhabitants, of which 6,135,970 (52.5%) are women^24^. It was divided into five Regional Health Coordinations and 25 Technical Health Supervisions and presented a territorial stratification (2015/2016 version) with 456 areas covered by Basic Health Units (BHUs), which are territories divided with the purpose of organizing the management of Primary Health Care^25^, as shown in Figure 1.

Between January 2009 and December 2016, the Mortality Information Improvement Program of the Mortality Information System (SIM/PRO-AIM) of the Epidemiology and Information Department, Municipal Health Department of São Paulo (CEInfo/SMS-SP), recorded 10,124 deaths from BC (10th Revision of the International Classification of Diseases — ICD-10: C50 to C50.9) and 2,116 deaths from CC (ICD-10: C53 to C53.9) in women aged 20 years and over in the city. These records were geocoded according to patients’ residence address, mainly through the Address Standardizer of the City of São Paulo. Addresses not found were geocoded by geolocation platforms that use Google Maps as a street database and by an application that has the Navteq^22^ street database.

**Figure 2.**
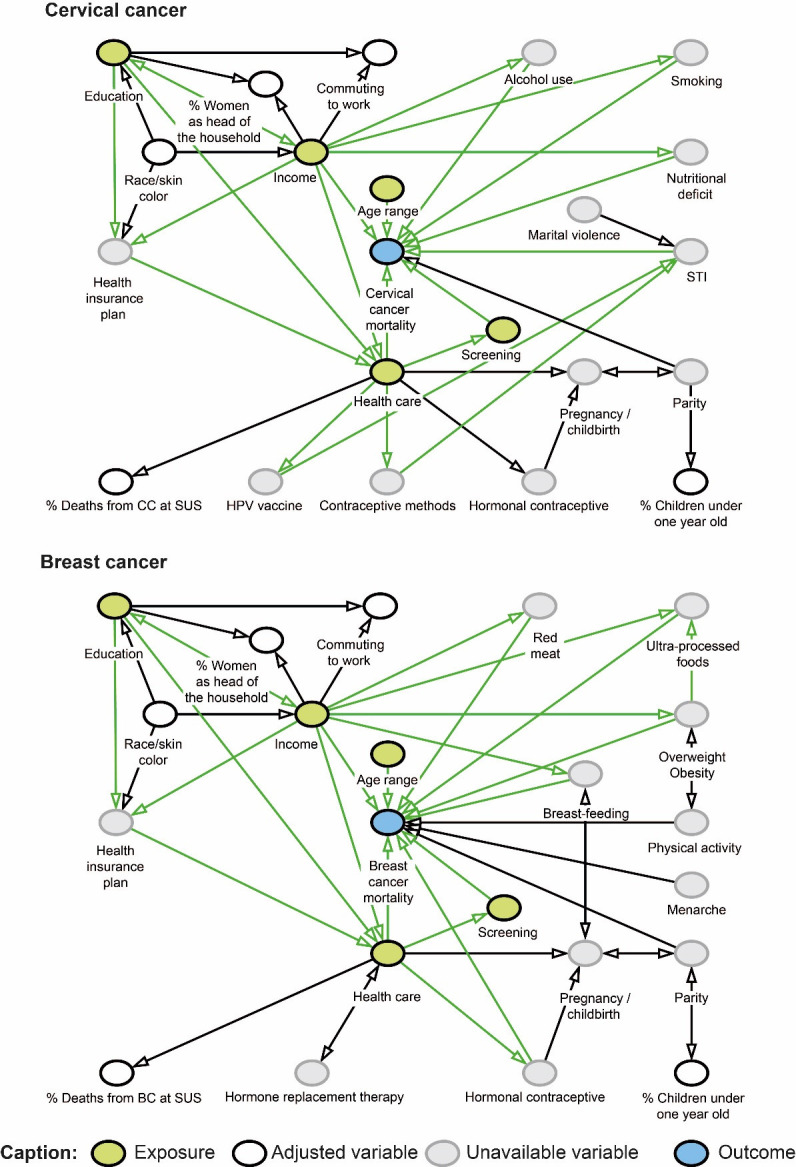
Conceptual theoretical models of factors associated with mortality from cervical and breast cancer represented by directed acyclic graphs.

The conceptual theoretical models of factors associated with deaths from BC and CC were represented by Directed Acyclic Graphs (DAG) (Figure 2). Spatial regression models were adjusted to check for association of deaths from BC and CC (dependent variables) with social, demographic, economic, educational, and care indicators (independent variables) (Supplementary Material 1).

Prepared by: Study Group on Spatial Analysis (GEANES/FSP/USP).

The digital grids of data of São Paulo’s coverage areas by BHUs were obtained from CEInfo/SMS-SP, and the independent variables used in the spatial regression analysis were acquired from the Brazilian Institute of Geography and Statistics (IBGE), SIM/PRO-AIM, Information System of Cervical Cancer (SISCOLO), Breast Cancer Information System (SISMAMA), Outpatient Information System (SIA) and National Registry of Health Establishments System (SCNES). The independent variables, made available in polygons (Census Sectors or Weighting Areas) by the IBGE, were recalculated for São Paulo’s coverage areas by BHUs using geographic operations, with the areas of these polygons considered as weights. The information provided by points (deaths and establishments) were aggregated into coverage areas through geographical intersection operation.

The number of deaths from BC and CC, grouped according to São Paulo’s coverage areas by BHUs, were modeled based on the Poisson probability distribution and through latent Bayesian Gaussian models^26^. Spatial autocorrelation was represented by structured and unstructured spatial random effects^27^, the Besag-York-Mollié model (BYM). The first one presents a conditional autoregressive (CAR) structure that smoothed the data based on the Queen-type contiguity neighborhood relation of order one. The second one is an independent and identically distributed random effect (iid) that modeled the uncorrelated noise. The BYM model was used with the proposed parameters^28^. The expected deaths from BC and CC were obtained, for all areas of coverage, by indirect standardization, based on specific rates by age groups. These values were considered in the modeling as offset, which made it possible to interpret the resulting coefficients as measures of relative risks (RR), having as parameters the mortality rates for the two cancers throughout the city during the study period for comparison.

Modeling was initially performed considering only intercepts and spatial random effects for each type of cancer. Before, however, an exploratory analysis was carried out to verify the existence of outliers and, when present, the independent variables were transformed by the cube root or logarithm (Supplementary Material 2). The independent variables were sorted into eight domains (Supplementary Material 3) and, for the ones presenting more than one variable, the independent variable that presented the lowest value based on the Deviance Information Criterion (DIC)^29^ was chosen in a bivariate modeling and considering spatial random effects.

With the set of independent variables for each condition, an exploratory analysis was carried out to assess collinearity through the variance inflation factor, considering values greater than or equal to 3 as cutoff point^30^. Then, a multiple regression modeling was performed, obtaining the posterior averages of RRs and respective 95% credible intervals (95%CI) adjusted for spatial autocorrelation^29^.

Modeling was performed using the Integrated Nested Laplace Approximation (INLA)^31^ approach. This is an efficient computational alternative to the Markov Chain Monte Carlo (MCMC) methods, especially when data has a spatial or space-temporal structure^26^. In the modeling, minimally informative priors were considered for fixed effects and priors with penalized complexity for random effects^32^. The analyses were performed in the R-INLA^31^ and INLAOutputs^33^ packages of the R program^34^.

## RESULTS

Of the total number of deaths from BC (10,124) and CC (2,116) registered, 10,066 (99.4%) and 2,101 (99.3%) were geocoded, respectively. The general rate of geocoding was 99.4%, and 92.1% of the records were geocoded at the exact point of the street and respective house number.

**Figure 3.**
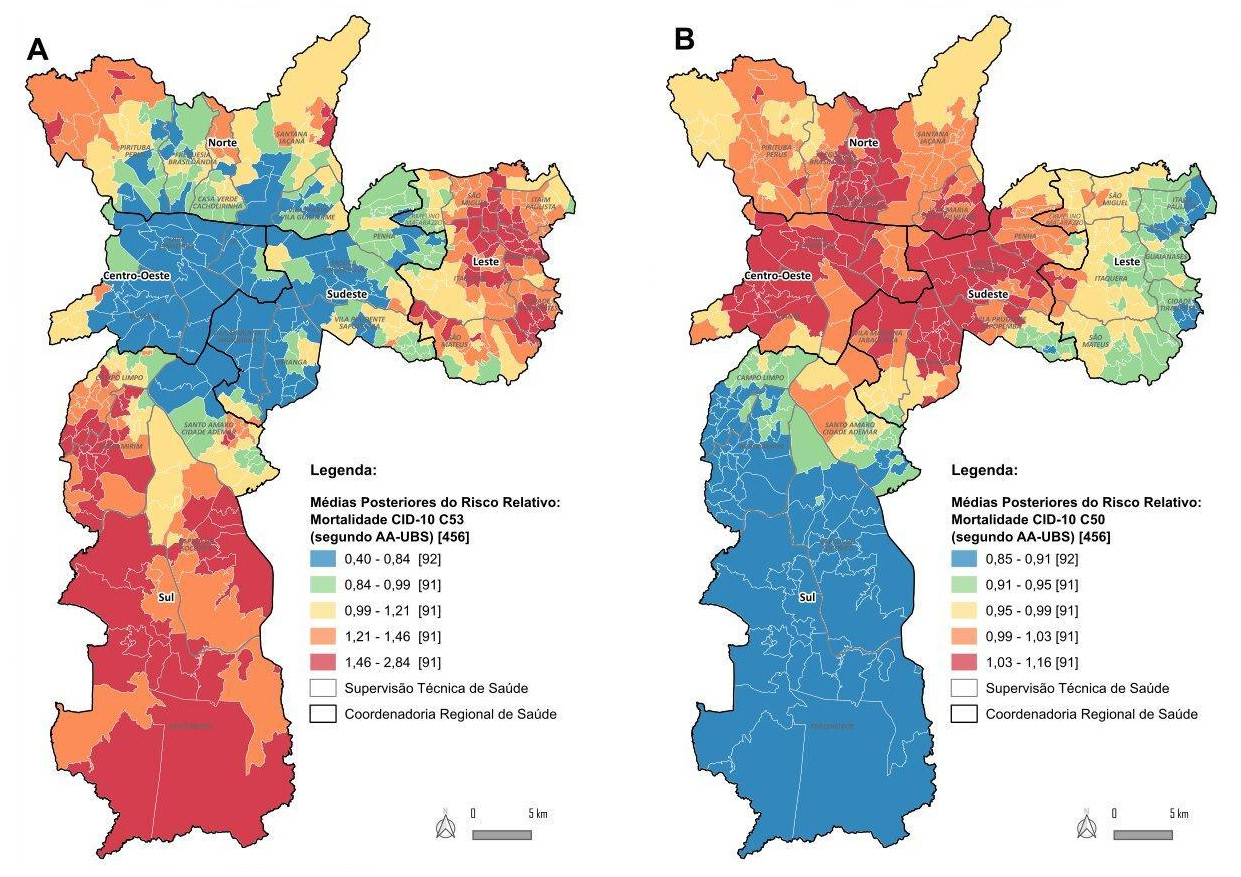
Posterior means of spatial relative risks of mortality from cervical cancer (A) and breast cancer (B) in women aged 20 years and over, according to areas covered by Basic Health Units. City of São Paulo, 2009 to 2016.

Source: PRO-AIM/SIM/CEInfo/SMS-SP, 2009-2016.

In Figure 3 shows the posterior averages of the RR predicted by the model for BC and the CC, obtained from the intercept modeling taking into account the structured and unstructured spatial random effects. Comparing the maps, both conditions presented opposite spatial patterns.

Mortality from BC in the city of São Paulo between 2009 and 2016, standardized by age group, showed a radial spatial pattern from the center of the city, that is, with higher predicted RR values in the central region, ranging from 1.03 to 1.16, and lower RR in peripheral regions, between 0.85 and 0.9. On the other hand, mortality from CC had higher values in peripheral regions, between 1.46 and 2.84, and lower values in the central region, from 0.40 to 0.84.

Table 1 shows the posterior means of RRs and respective 95%CI of each independent variable, adjusted for the spatial autocorrelation of mortality from BC and CC, respectively.

**Table 1.** Posterior means of spatial relative risks and credible intervals (95%CI) of factors associated with mortality from cervical and breast cancer in women aged 20 years and over, according to areas covered by Basic Health Units (2015/2016 version). City of São Paulo, 2009-2016.

Breast cancer	RR	95%CI
Proportion (%) of employed people in the reference week with usual time of commuting for work from more than 1 hour to 2 hours	0.97	(0.93–1.00)
Proportion (%) of women as head of the household	0.97	(0.94–0.99)
Ratio (%) of children under 1 year of age related to the female population aged 15 to 49 years	0.99	(0.96–1.02)
Proportion (%) of deaths from breast cancer occurring in private health institutions	1.04	(1.00–1.07)
**Cervical cancer**	**RR**	**95%CI**
Proportion (%) of employed people in the reference week with usual time of commuting for work from more than half an hour to 1 hour	0.92	(0.87–0.98)
Household income (private household) per capita up to 3 minimum wages in July 2010	1.27	(1.18–1.37)
Proportion (%) of women as head of the household	1.01	(0.96–1.07)
Ratio (%) of children under 1 year of age related to the female population aged 15 to 49 years	1.09	(1.01–1.18)
Proportion (%) of deaths from cervical cancer that occurred in public health institutions/associated with the Unified Health System (SUS)	0.98	(0.90–1.06)

RR: relative risk; CI: credible interval. Source: PRO-AIM/SIM/CEInfo/SMS-SP, 2009 to 2016.

As to mortality from BC, three variables showed a statistically significant association. The “proportion (%) of employed people in the reference week with a usual time of commuting from work of more than one hour to two hours” and the “proportion (%) of women as head of the household” were negatively associated with the outcome. The variation of one standard deviation in these two variables corresponded to a 3% decrease in mortality. The variable “proportion (%) of deaths from BC in private health institutions” was positively associated with the outcome, and the variation of one standard deviation in this variable corresponded to a 4% increase in mortality.

As for CC mortality, three variables showed a statistically significant association. The variable “proportion (%) of employed people in the reference week with usual time of commuting to work from more than half an hour to one hour” had a negative association, and the variation of one standard deviation corresponded to an 8% decrease in mortality. The variables “household income (private household) per capita up to 3 minimum wages in July 2010” and “ratio (%) of children under one year of age related to the female population aged 15 to 49 years” showed a positive association. The variation of one standard deviation in these two variables corresponded to an increase in mortality by 27% and 9%, respectively.

## DISCUSSION

Mortality rates from breast cancer and cervical cancer showed opposite spatial patterns, associated with the socioeconomic conditions of the areas covered by BHUs in São Paulo.

Regarding BC, the variables with a significant effect on mortality from this disease show that areas with a lower socioeconomic level have a lower risk of mortality from this cancer, while areas with a better socioeconomic level have a higher risk. Several studies discuss the positive association between mortality from BC and better socioeconomic levels^23,35–37^.

A study by Pereira and Schwanen^38^ had already shown that, in Brazil, the poorest population tends to spend more time commuting from home to work than the richest; in our study, commuting time of one to two hours to work was negatively associated with mortality from BC. Another inversely associated factor was the proportion of women as head of the household. An analysis of the Brazilian demographic census showed that, in most households whose heads are women, there is no spouse/partner; and almost half of them (45.5%) received an income of up to one minimum wage in 1991, a proportion (of women with such low incomes as head of a household) that decreased to 33.3% in 2000^39^.

Regarding mortality from CC, the proportion of children under one year of age related to the female population in childbearing age was associated with the outcome. This coincides with the findings of another Brazilian study, which found a positive correlation between fertility and mortality from this disease^36^. However, this indicator is also related to socioeconomic level, since, in Brazil, women with fewer years of schooling and lower per capita income have higher fertility rates^40^.

These findings are in agreement with those observed in the study carried out at the city of São Paulo^23^, which found that mortality from BC is higher in populations with better socioeconomic status, while mortality from CC occurs more frequently in economically disadvantaged populations. However, worldwide trend studies show that higher-income countries have higher rates of BC occurrence, but not mortality^2^.

Regarding health care variables, only the “proportion (%) of deaths from breast cancer that occurred in private health institutions” was associated with mortality from BC — perhaps due to the lack of data transparency from private services and the lack of data on coverage of health plans in the unit of analysis. According to ISA Capital^41^, the proportion of women who reported having a health insurance plan in the city of São Paulo was 43.4%, with a significant difference regarding level of education, being higher for those who reported 12 or more years of schooling (68, 5%); and for those with a per capita family income of five or more minimum wages (72.5%). Thus, given the relevance of this indicator, working on policies that encourage the qualified dissemination of screening tests by supplementary health is necessary, with a view to a better assessment of the effectiveness and efficiency of these prevention measures.

A recent study conducted in Brazil reported that mortality from BC had a significant negative association with the level of public health expenditure^42^. However, caution is needed in this assessment. Currently, BC screening measures are mostly based on mammography, which is considered the gold standard compared to other imaging methods. However, several recent studies have linked the increase in this type of screening with greater morbidity from BC due to overdiagnosis and overtreatment^37,43–46^. According to Tesser and d’Ávila^47^, who question the recommendation of BC screening, overdiagnosis is a hypothesis to explain the mismatch between the sustained increase in the incidence of cancers after the initiation of screenings, disproportionate to the little or no change in mortality (compared to unscreened populations) and morbidity, as there is no proportional reduction in advanced forms of cancer. If there is a sustained increase in incidence at the expense of the early stages, without a proportional reduction in mortality and/or in advanced forms, it is very likely that the new post-screening diagnoses were not of diseases that would lead to death, as expected, because, if they were, a decrease in the incidence of advanced forms and mortality would be expected.

With regard to CC, in addition to the preventive Pap smear in 2014, the Ministry of Health included regular vaccination against HPV in the calendar^48^. Nevertheless, some studies still reflect the lack of knowledge and the socioeconomic and racial inequality in the access to actions of cancer prevention programs^49–52^. Considering that the vaccine is in fact effective, its result in the incidence or mortality of this cancer can only be verified in years or even decades.

The variables related to race/skin color were also not related to mortality from both cancer types; self-declared information, which is unfeasible at death, can generate bias when interpreting data collected^53^. The illiteracy rate in the elderly population was also not associated with any of the diseases, probably due to the low proportion of illiterate elderly in the city of São Paulo — 5.9%^54^.

A study conducted in Paraná, Brazil, sought to spatially assess the socioeconomic disparities in access to health related to mortality from BC and reported results that agree with ours. The authors explain that better socioeconomic conditions seem to be related to variables that influence mortality from this type of cancer. A high score of accessibility to oncology services (radiotherapy and chemotherapy) was positively correlated with mortality rates^55^. Considering the incidence of BC, a spatial analysis with smoothed incidence rates in the region of Iran, using spatial scanning statistics, showed that the incidence is a health problem in rich areas with higher levels of education and higher expenditures on health actions. Women living in these areas of better socioeconomic status were found to have higher expenditures on health care activities such as screening exams, resulting in a more frequent diagnosis of BC. In addition, they have easier access to cancer treatment centers and adjuvant therapies and are, therefore, likely to have better survival rates^56^.

However, American studies found a pattern of mortality from BC that differed from our findings. A national-scale study^57^ analyzed the geographic variation in incidence and mortality of female BC, detecting several significant spatial clusters that persisted even after adjusting for risk factors. Another study conducted in the United States^58^ evaluated geographic, racial and ethnic variations associated with mortality from BC among women living in 3,108 contiguous municipalities, from 2000 to 2015. Overall, mortality rates were higher in municipalities with high proportion of non-Hispanic black residents, low education and income, among others. However, the evaluation of the spatial variation of BC-specific mortality in Louisiana showed that higher mortality was associated with low income, worse socioeconomic status, poor access to and quality of health care, availability of fresh food, rurality and certain occupations in certain industries^59^.

With regard to CC, improving early detection and providing timely treatment is necessary to alleviate the burden of the disease. Population-based research in Costa Rica used data from the National Tumor Registry and assessed inequalities in the incidence of the disease. The results indicated a modest decrease in inequality between 1980 and 2010, given that the female population under 40 years of age had access to benefits from a national prevention program that was not experienced by the female population above this age group. Thus, CC cases dropped only among the youngest population, increasing the inequality in incidence^60^.

A study conducted in Baltimore evaluated the neighborhood correlation and spatial distribution of BC, CC, and colorectal cancer from 2000 to 2010 and found remarkable geographic variation by cancer type. CC was often diagnosed at younger ages when compared to BC, which was more impacted by the increasing age of residents^61^. These results are in line with our study, which detected a higher risk of CC mortality in more peripheral areas of the city, characterized by a higher concentration of young people; and higher mortality risk for BC in more central areas, where mainly the older population resides.

The higher mortality from CC in areas of lower socioeconomic status in São Paulo translates into poor access to health services and, consequently, lower frequency of screening for the detection of cancer in the initial phase, which is an effective measure to reduce mortality^62^. The efficiency of the screening program could also be observed in a city in Romania, a country with one of the highest CC incidence and mortality rates in Europe^63^. After the implementation of the Pap smear screening program in 2012, there was a reduction in the diagnosis of cases in stage IV, as well as an increase in the incidence of in situ cancer^64^. The study conducted in Costa Rica^60^ also provided evidence that inequality in CC incidence over three decades was associated with underutilization of screening methods in certain regions of the country.

A limitation of the study was the use of data available, but outdated, from the 2010 Demographic Census. Another limitation was the lack of access to the number of procedures performed in private institutions, which makes it difficult to calculate the coverage of preventive exams. The availability of information regarding the number of beneficiaries in the recommended age groups for screening would help to establish with greater precision the effectiveness of measures adopted.

In order to improve the analysis of the health situation in large urban centers, incorporating spatial analysis of data at the intra-municipal scale is recommended. This study puts the spotlight on issues related to women’s health in a localized level, as it allows one to view the phenomenon according to areas covered by BHUs, a scale closer to reality.

## RESEARCH ETHICS COMMITTEE IDENTIFICATION

This study is linked to the Study Group on Spatial Analysis (GEANES) of the School of Public Health of Universidade de São Paulo (FSP-USP), and was submitted to its Research Ethics Committee under Certificate of Presentation for Ethical Assessment (CAAE) 76049317.7.0000.5421; approved on December 4, 2017, according to Opinion No. 2,412,427; as well as to the Ethics Committee of the Municipal Health Department of São Paulo (SMS-SP), under CAAE 76049317.7.3001.0086 and approved on January 16, 2018, according to Opinion No. 2,467,549.
